# The human placenta as a model for training and research in mechanical thrombectomy: Clarifications and use of the chorionic plate veins

**DOI:** 10.3389/fneur.2022.925763

**Published:** 2022-09-20

**Authors:** Julien Burel, Jonathan Cornacchini, Matthieu Garnier, Sophie Patrier, Albane Guigné, Emmanuel Gerardin, Chrysanthi Papagiannaki, Nader Sourour, Eimad Shotar, Kévin Premat, Claire Laporte, Frédéric Clarençon

**Affiliations:** ^1^Department of Radiology, Rouen University Hospital, Rouen, France; ^2^GRC BioFast, Sorbonne University, Paris, France; ^3^Department of Pathology, Rouen University Hospital, Rouen, France; ^4^Forensic Department, Rouen University Hospital, Rouen, France; ^5^Department of Neuroradiology, Pitié-Salpêtrière Hospital, APHP, Sorbonne University, Paris, France

**Keywords:** stroke, mechanical thrombectomy, placenta, vascular model, interventional neuroradiology

## Abstract

Indications for mechanical thrombectomy in acute ischemic stroke are increasing, resulting in the continuous development of new devices and techniques. Therefore, there is a need for a realistic testing and training environment that offers the opportunity to practice different procedures and test the latest devices. Some authors have described the use of the human placenta as a model for neurointerventional surgery, with striking similarities to real-life conditions. This model has many advantages, including its relatively low cost and minimal infrastructure requirements, with fewer ethical concerns compared to animal models. So far, some preparation and set-up details were missing, and only arteries from the chorionic plate were used. This article provides the necessary clarifications and a mapping of the chorionic plate veins, so that the use of this model, which is particularly well suited for mechanical thrombectomy, can be as easy and wide as possible. A video explaining how to prepare the model is provided.

## Introduction

Randomized controlled trials on acute ischemic stroke due to large vessel occlusion established the superiority of mechanical thrombectomy (MT) in addition to the best medical management, including intravenous thrombolysis, over the best medical management alone within 6 h from symptom onset (1–6). More recent trials demonstrated that the time window for MT can be extended up to 16 (7) or 24 h (8) from the last time the patient was known to be well, when the selection is based on neuroimaging evaluation showing a salvageable penumbra (7) or a mismatch between clinical deficit and infarct size (8). Different techniques are currently used to perform these procedures, including stent retriever alone, contact aspiration alone, and combined techniques (using in different fashions stent retriever and aspiration catheter at the same time). However, there is no consensus on the optimal method for thrombectomy. Moreover, due to all these successes, there is a continuous development of novel therapeutic approaches. Hence, there is the need for a realistic testing and training environment with the possibility of practicing different procedures and improving neurointerventional skills. Available training setups include computer-based simulations, in *vitro* training using generalized or patient-specific vascular models, and animal-based training, such as swine or rabbit models (9, 10). While each model has certain advantages and drawbacks, it is difficult to reproduce all the haptic qualities necessary for these procedures using virtual simulators or animal model (11). It is therefore necessary to further improve existing models and to develop new models for research and training.

The human placenta (HP) is used as a vascular model for interventional neuroradiology. The HP model has many advantages, including its relatively low cost, minimal infrastructure requirements, ease of preparation and set-up, with fewer ethical concerns compared to animal models. Kwok et al. (9) conducted in 2014 some experimental studies, including a simulation of intra-arterial thrombolysis and a simulation of MT, with a comparison of different devices. However, the sample sizes were too small to perform statistical analyses. They reported that this model has striking similarities to real-life conditions. Nevertheless, some improvements are needed to better match real patient situations and to expand its use in research. Indeed, articles about the HP in neurointerventional surgery all report the use of CPAs to simulate intracranial arteries (9, 11, 12), whereas the chorionic plate arteries (CPAs) cross the chorionic plate veins (CPVs) above, unlike the intracranial arteries with the veins; and the umbilical arteries are much more difficult to catheterize than the veins. This article therefore explores the possibility of using the CPVs to simulate intracranial arteries and conduct research on MT.

## Anatomy, histology and preparation method

### Obtaining placentas

Fresh HP is relatively easy to obtain from hospitals with obstetric services. Written consent for donation of placenta for research and training in neurointerventional surgery is obtained from the mother before the baby is delivered. These placentas can be kept under refrigeration for approximately 2 weeks. However, it should be noted that the fresher the placenta is, the more faithful it will be to the actual practice conditions, and the tighter the artery-vein circuit will be.

### Ethical statement

In most countries, including France, the placenta is considered as a human waste (like hair or nails). To avoid any confusion, all the women signed an informed consent form clearly stipulating that the placenta will be used only for training and research purposes and that no genetic research would be performed on the specimen.

### Anatomy

**Figure 1:**
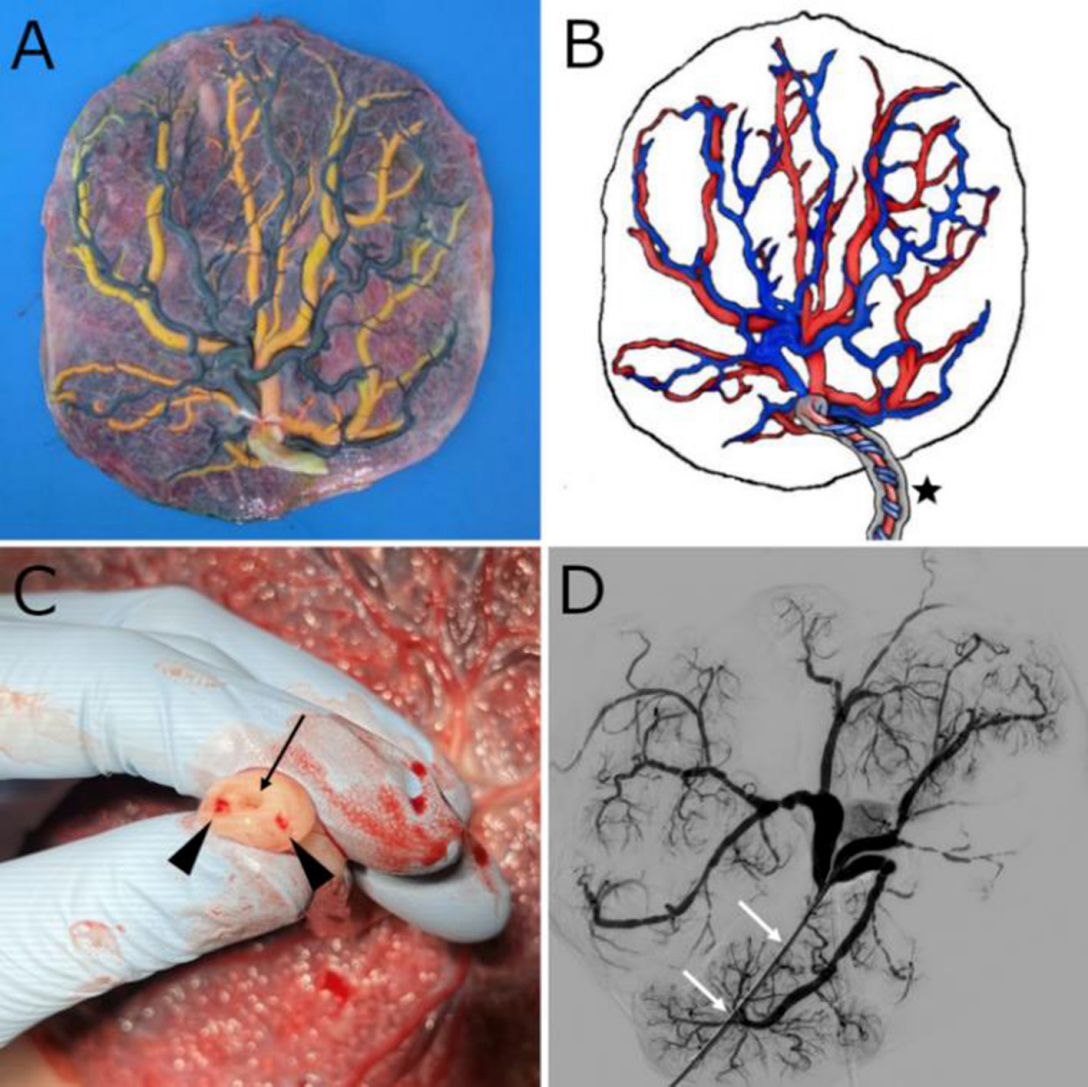
**(A)** Fresh placenta after removal of the amniotic sac and injection of color-dye into the chorionic plate vessels. Yellow vessels correspond to veins and dark green ones to arteries. **(B)** Schematic representation of a human placenta. The umbilical cord (black star) contains a vein and two arteries that spiral around it, buried within Wharton's jelly. The veins, which carry oxygenated blood, are shown in red, and the arteries in blue. **(C)** Umbilical cord cross-section with two umbilical arteries (black arrowheads) and a single umbilical vein (black arrow). **(D)** 2D-Angiographic appearance of the chorionic plate vein network after injection of contrast medium through an introducer sheath placed at the junction between the umbilical vein and the chorionic plate veins. The white arrows show the introducer sheath.

A normal full-term HP is a circular discoidal organ with a diameter of about 22 cm, a central thickness of 2.5 cm, and an average weight of 470 g (13). The umbilical cord contains one vein (the umbilical vein) and two arteries (the umbilical arteries) surrounded by Wharton's jelly (see Figure 1). The umbilical vein carries oxygenated, nutrient-rich blood from the placenta to the fetus, and the umbilical arteries carry deoxygenated, nutrient-depleted blood from the fetus to the placenta (14). The umbilical arteries spiral around the umbilical vein. The umbilical cord most often inserts slightly eccentrically into the chorionic plate. The Hyrtl anastomosis is a common connection between the umbilical arteries near the cord insertion in most HPs. It has two main roles: safety valve (shunt), in case of partial compression of the placenta during uterine contractions or occlusion of one umbilical artery; pressure stabilizer between the umbilical arteries, when one of the arteries conducts a smaller blood flow into the placenta and a relatively smaller pressure gradient is developed, rebuilding the pressure gradients in the affected artery, and redistributing blood flow from the unaffected artery to the affected one to improve placental perfusion (15). Hyrtl anastomosis can be either a single connecting vessel or a fusion between the umbilical arteries. Most of the anastomoses (up to 90%) are of a single connecting tube, which may be transverse or oblique to the arteries (16).

The chorionic plate represents the fetal surface of the placenta, which is covered by the amnion. The amnion is only weakly attached to the chorion and can easily be removed from the delivered placenta. The chorion contains the chorionic vessels which are in continuity with the umbilical cord vessels. The chorionic plate arteries (CPAs) branches derive from the two umbilical arteries and form a disperse pattern where each artery divide in two or more branches, with gradually diminishing diameter, until their final branches, which supply the villous trees. The CPVs are direct extensions of the veins of the villous trees and usually cross the CPAs below. The CPVs give rise to the single umbilical vein (13).

Regarding CPAs, Bekov (17) defined the first-order segments as those diverging from the umbilical cord. The third-order segments are the end segments on the fetal surface that dive into the chorion to provide circulation into distinct cotyledons. The second-order segments are located between the first and third segments. Belykh et al. (18) provided data regarding CPAs lengths and diameters and a comparison with intracranial arteries. The first-order segments have a mean length of 28.8 ± 9.9 mm and a mean diameter of 6.5 ± 1.4 mm. The second division has a mean length of 35.9 ± 15.3 mm and a mean diameter of 3.4 ± 0.7 mm; these diameters are comparable to that of the first segment of a middle cerebral artery (M1 segment), an intracranial internal carotid artery (ICA) or a vertebral artery. The third-order segments have a mean length of 29.9 ± 10 mm and a mean diameter of 1.7 ± 0.4 mm; these diameters are comparable to that of an anterior cerebral artery, a posterior inferior cerebellar artery (PICA) or M2 to M4 segments. To the best of our knowledge, there is no study that maps the average lengths and diameters of the different segments of CPVs.

The basal plate represents the maternal surface of the placenta. It is an artificial surface, which emerged from the separation of the placenta from the uterine wall during delivery. A system of flat grooves or deeper clefts subdivides the basal plate into 10–40 slightly elevated regions called cotyledons, each of which consists of a main stem villus and all its branches (13). Because the basal plate is not used in neurointerventional training or research, its description will not be further detailed.

### Preparation

A strict transmissible disease protection protocol must be observed in all procedures. The first step is to remove the peripheral membranes with scissors and to peel off the portion of the amnion that adheres to the chorionic plate from the periphery to the umbilical cord, exposing the chorionic plate. Next, the blood adhering to both sides of the placenta should be removed with water or saline. The umbilical cord should be shortened by a clean cut with a scalpel to a length of 3–5 cm in order to facilitate future catheterization of the umbilical vessels, especially the arteries, which spiral around the vein. The two umbilical arteries and the umbilical vein can now be easily identified at the umbilical cord cross-section (Figure 1C).

The placenta is placed in a tray, which will be slightly inclined after preparation, to facilitate the emptying of fluid and contrast medium that extravasates out of the delicate capillary system, thus keeping the radiological field clear. The guide wire provided with a 6F introducer sheath is placed in the umbilical vein up to the CPVs, allowing then to position the introducer sheath with its dilator on this guide wire. The same strategy is used to catheterize each of the umbilical arteries up to the chorionic plate, with two particularities: the primary use of size 4–5F material may be useful to facilitate catheterization in these smaller vessels; rotating the cord around the venous introducer sheath is often useful to decrease the tortuosity of the umbilical arteries. The 4–5F introducers can be exchanged for 6–7F introducers for arteries and an exchange with 7–8F material can be done for the vein, to allow the introduction of larger caliber catheters depending on the context of use. A suture should now be placed around each umbilical vessel to avoid fluid reflux into them, another method being the use of clamps. At this stage, a 100- to 140-mmHg pressure bag is used to deliver a heparinized saline solution *via* an IV line into each arterial introducer (or in the venous introducer if the vessels used later for research or training will be the veins), dilating the vessels and removing the intraluminal clots. Note that digital pressures can help remove clots. Another IV line is connected to the venous introducer (or to each arterial introducer, if the anti-physiological direction is used), and its other end is placed in a tray, as the end of the circuit (placing a few gauze sponges in the bottom of this tray allows to mop up the liquid). Digital subtraction angiography (DSA) can now be practiced being as close as possible to the real conditions of intervention.

Note that one of the advantages of the HP model is the semi-transparent nature of the chorionic plate, which allows direct observation of changes within the blood vessels, the behavior of catheters, devices, clots, etc.

### Histopathology and immunohistochemistry

For histopathological assessment, areas of the placenta that include CPAs and CPVs can be obtained to allow routine processing in cassettes. Samples are routinely fixed for at least 48–72 h in neutral buffered formalin before processing and embedding into paraffin blocks, from which histological sections of 3–5 μm can be obtained. Once in blocks, samples may be stored indefinitely at room temperature (19).

Unlike cerebral arteries, CPAs do not have an internal elastic lamina or elastic fibers in the media (18); therefore, these elements cannot be used for research purposes. However, because the endothelium of CPAs is positive for CD31 staining (9), the change in endothelium lining can be studied easily. The endothelial response to different devices or drugs can then be examined, which opens the door to many studies, such as the comparison between different MT devices or the comparison between different MT techniques.

## Using CPVs as intracranial arteries

An improvement to the HP model could be its anti-physiological use, i.e., using CPVs as intracranial arteries. Indeed, CPAs are most often used to simulate intracranial arteries in neurointerventional surgery (9, 11, 12), whereas umbilical arteries are more difficult to catheterize than the vein, which is more straight and has a greater diameter. Furthermore, unlike intracranial vessels which have markedly different properties, histology, and vessel wall thickness between arteries and veins, due to different pressure requirements, chorionic plate vessels show far fewer disparities. Finally, this larger diameter, which allows the placement of larger introducers, will make it possible to use other catheters, capable for example of simultaneously accommodating two microcatheters. It is therefore necessary to map the lengths and diameters of the CPVs, and to verify that the endothelium of the placental veins is correctly labeled by CD31 (or CD34).

### Methods

After written consent from the mothers, 6 placentas were prepared with the above-mentioned method (Supplementary Video 1), allowing the acquisition of standard anteroposterior 2D-projections and 3D-rotational angiography, after injection of iodinated contrast medium (6 mL with a velocity of 3 mL/s for 2D-projections and 15 mL with a velocity of 3 mL/s for 3D-rotational angiography), with a monoplane angiographic system (Azurion; Philips Healthcare, Best, the Netherlands). The lengths and diameters of the chorionic plate venous segments were measured, allowing calculation of the mean measurements, with standard deviation. Measurements were made by one interventional neuroradiologist on the 3D-angiograms, on all segments well opacified by the contrast medium.

On another placenta, in which color-dye was injected (the one shown in Figure 1), immunohistochemical analyses were performed to verify that the venous endothelium is labeled by CD31. The placenta was fixed for 7 days in neutral buffered formalin before processing and embedding into paraffin blocks, from which histological sections were obtained, and immunostainings with CD31 were performed. Immunohistochemical analyses were performed by one perinatal pathologist.

### Results

**Figure 2:**
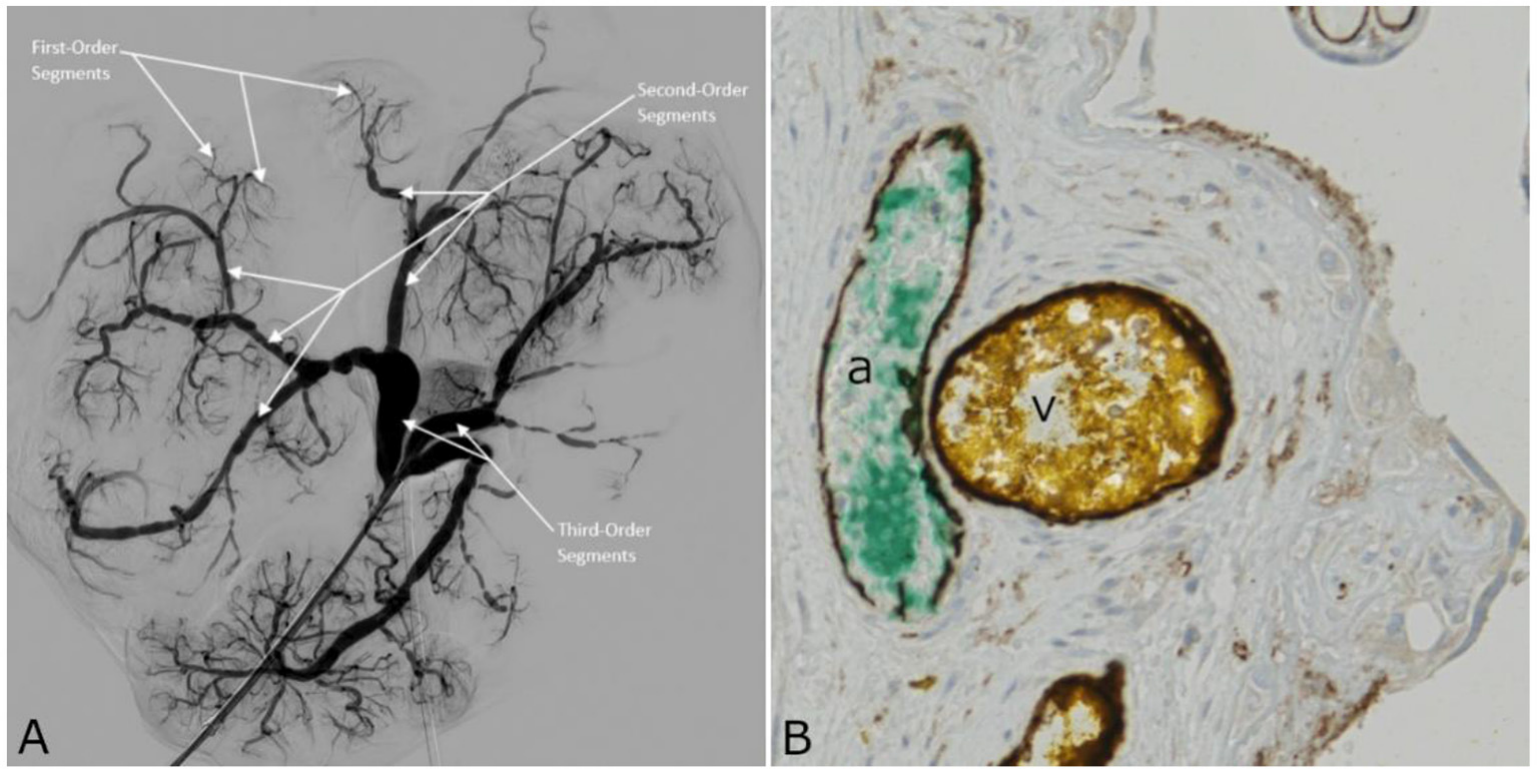
**(A)** 2D-Angiography of the chorionic plate veins with white arrows indicating first-, second-, and third-order segments. **(B)** Cross sectional image of the placenta shown in Figure 1A, obtained by light microscopy. The tissue sample was stained with CD31 for endothelium. Both CPA (a) and CPV (v) endothelia are CD31 positive, and appear in dark brown.

By analogy with Bekov's classification for CPAs (17), it can be defined (Figure 2): the first-order segments of the CPVs, those directly following the veins of the villous trees; the second-order segments, between the first and third segments; the third-order segments, joining the umbilical vein. Table 1 shows the average diameters and lengths of the first-, second-, and third-order venous segments. Figure 2B shows the immunohistochemical positivity of the arterial and venous vascular endothelium to CD31.

**Table 1:** Average diameters (mm) and lengths (mm) of the first-, second-, and third-order venous segments.

**Segments**	**Mean Diameter ±SD (range)**	**Mean Length ±SD (range)**
Third-order	11.6 ± 3.6 (7.4–17.9)	33.6 ± 8.8 (21.8–46)
Second-order	3.7 ± 1.1 (2.3–5.7)	28.3 ± 10.7 (15.1–49.7)
First-Order	1.3 ± 0.5 (0.7–2.1)	8.2 ± 2.3 (3.5–13.9)

## Discussion

The HP is a useful vascular model for training and research in MT. One of its advantages is the ease of implementation in any hospital with a maternity ward and an angiographic system, at a very low cost. Unlike computer-based and silicone models, it can be used to study the endothelial response to drugs and devices used in MT. Another advantage is that a single HP has several vessels that can all be used for simulation and reproduce various endovascular anatomical situations. Furthermore, there are fewer ethical issues compared to animal models, and the equipment and personnel requirements are much lower. Ultimately, a single placenta can be used for several training sessions, as it keeps very well for up to 2 weeks.

Although the HP model cannot be used in longitudinal follow-up studies like animal models (10), it could be used to study immediate hemodynamic changes induced by different devices, such as stents for “intracranial” stenosis. This could be done in all imaging modalities and more easily than with silicone models. The absence of great vessels and supra-aortic trunks analogs in the HP model can be compensated by the combined use of HP and other vascular models, such as silicone models.

So far, papers referring to the use of placenta in neurointerventional surgery all reported the use of CPAs to simulate intracranial arteries (9, 11, 12). This study demonstrates that CPVs can also be used thanks to their anatomy and histology, which are very similar to those of arteries. The following advantages have been observed: CPVs cross the CPAs below, just as intracranial arteries do with respect to veins; and the umbilical vein is much easier to catheterize initially (including with larger catheters), and can be the only vessel catheterized in case of difficulties in catheterizing arteries that are too tortuous (it would, however, implies higher fluid losses). However, it is preferable, if possible, to catheterize both the umbilical arteries and the umbilical vein, thus obtaining a closed vascular model and a larger number of vessels to use for research or training. Indeed, it is possible to change the direction of fluid flow during experiments, as both CPAs and CPVs can simulate intracranial vessels. Second-order venous segments of the chorionic plate, with a mean diameter of 3.7 ± 1.1 mm (range = 2.3–5.7 mm), are ideally suited for training and research in MT, because these values correspond to those of the large arterial trunks for which MT is performed. In addition, third-order venous segments, with an average diameter of 11.6 ± 3.6 mm (range = 7.4–17.9 mm), can sometimes be used to train for cervical carotid stenting when the length is sufficient [33.6 ± 8.8 mm (range = 21.8–46 mm)].

Currently, interventional neuroradiologists who perform procedures on *ex vivo* vascular models, including HP, use water or saline solution to represent blood. Unlike these fluids, blood is a shear-thinning fluid. As a consequence, it makes the simulation less realistic because it lacks tactile feedback. The best blood mimicking fluid must reproduce the physical properties and rheology of blood. This would allow tactile sensations similar to those of real patients, and the behavior of devices and drugs would be more accurate. In addition, if the fluid can be studied realistically in Doppler, it would be a significant improvement. Blood itself might seem like the best fluid to use. However, there are several limitations in using blood and its components. It is a potential biohazard and precautions must be taken to minimize this risk. In addition, the shelf life of blood is limited, and *in vitro* erythrocytes are easily damaged. Moreover, the rheological properties of blood are likely to be different at room temperature than at 37°C (20). A 60/40 (by volume) water–glycerol mixture is one of the most widely used blood-analog solutions for use with vascular flow models (21). It has a viscosity of 3.8 ± 0.08 × 10^−6^ m^2^/s and a density of 1090 ± 15 kg/m^3^ (22), which is comparable to human blood. This water-glycerol solution is an inexpensive option that can be used with the HP model to improve tactile feedback.

Vessel pulsatility in the HP model can be simulated by using a programmable pulsatile perfusion pump, generating varying pressures and flow volumes at predetermined intervals. Many pumps of this type already exist and are commercially available, the pumps used with silicone models being compatible with the HP model. For preclinical evaluation and development of MT devices, and for training in interventional techniques with these devices, clot analogs that are as close as possible to the clots that cause stroke in humans should be used. Such clots with different fibrin and red blood cells compositions have been developed as part of the Neuro Thromboembolic Initiative of Neuravi (Cerenovus, Galway, Ireland) (23).

To conclude, HP may provide a low-cost training and research model for MT. The use of the CPVs seems to facilitate model preparation and may open new insights. The immunohistochemical positivity of the arterial and venous endothelium to CD31 opens the door to fundamental research projects. Further studies are however warranted to validate this model.

## Data availability statement

The raw data supporting the conclusions of this article will be made available by the authors, without undue reservation.

## Ethics statement

Written consent for donation of placenta from the mothers was obtained. An explanation of what interventional neuroradiology is and how the placenta would be used was given to the mothers in a printed format.

## Author contributions

JB, JC, SP, and FC: conception and design. JB, MG, AG, EG, CP, NS, ES, KP, CL, and FC: acquisition of data. JB, MG, SP, AG, EG, CP, NS, ES, KP, CL, and FC: analysis and interpretation of data. JB, JC, and FC: draft of the manuscript. JB, JC, MG, SP, AG, EG, CP, NS, ES, KP, CL, and FC: revision and final approval. All authors contributed to the article and approved the submitted version.

## Conflict of interest

Author NS reports a conflict of interest with Medtronic, Balt Extrusion, Microvention (consultant), and Stock/Stock Options: Medina. Author FC reports a conflict of interest with Medtronic, Guerbet, Balt Extrusion (payment for readings), and Codman Neurovascular (core lab). The remaining authors declare that the research was conducted in the absence of any commercial or financial relationships that could be construed as a potential conflict of interest.

## Publisher's note

All claims expressed in this article are solely those of the authors and do not necessarily represent those of their affiliated organizations, or those of the publisher, the editors and the reviewers. Any product that may be evaluated in this article, or claim that may be made by its manufacturer, is not guaranteed or endorsed by the publisher.

## Abbreviations

MT: Mechanical Thrombectomy
HP: Human Placenta
CPA: Chorionic Plate Artery
CPV: Chorionic Plate Vein
M1 segment: First segment of Middle cerebral artery
ICA: Internal Carotid Artery
PICA: Posterior Inferior Cerebellar Artery
DSA: Digital Subtraction Angiography.
